# Disrupting the Hsp90–Cdc37 axis: a selective strategy for targeting oncogenic kinases in cancer

**DOI:** 10.1039/d5ra03137k

**Published:** 2025-06-09

**Authors:** Emadeldin M. Kamel, Mohamed A. M. Ali, Ahmed A. Allam, Noha A. Ahmed, Faris F. Aba Alkhayl, Al Mokhtar Lamsabhi

**Affiliations:** a Chemistry Department, Faculty of Science, Beni-Suef University Beni-Suef 62514 Egypt emad.abdelhameed@science.bsu.edu.eg; b Department of Biology, College of Science, Imam Mohammad Ibn Saud Islamic University (IMSIU) Riyadh 11623 Saudi Arabia; c Physiology Division, Zoology Department, Faculty of Science, Beni-Suef University P.O. Box 62521 Beni-Suef Egypt; d Department of Medical Laboratories, College of Applied Medical Sciences, Qassim University Buraydah 51452 Saudi Arabia; e Departamento de Química and Institute for Advanced Research in Chemical Science (IAdChem), Facultad de Ciencias, Módulo 13, Universidad Autónoma de Madrid 28049 Madrid Spain

## Abstract

Heat shock protein 90 (Hsp90) is a crucial molecular chaperone responsible for the maturation and stabilization of a wide range of client proteins, many of which are key drivers of oncogenic signaling. While traditional Hsp90 inhibitors targeting its ATPase activity have demonstrated antitumor potential, their clinical progress has been limited by issues such as low selectivity, toxicity, and the induction of cytoprotective heat shock responses. An alternative strategy focuses on disrupting the specific protein–protein interaction between Hsp90 and its kinase-specific co-chaperone, cell division cycle 37 (Cdc37), thereby selectively destabilizing oncogenic kinases without broadly impairing chaperone function. This review discusses the structural insights into the Hsp90–Cdc37 interface, recent advances in the discovery of small molecule inhibitors, peptides, peptidomimetics, and natural products such as celastrol, platycodin D, and withaferin A that effectively disrupt this interaction. Mechanistic studies reveal that disruption leads to targeted degradation of kinase clients, inhibition of key survival pathways including AKT and ERK signaling, induction of apoptosis, and sensitization to other therapeutic agents, all while minimizing activation of the heat shock response. Despite challenges related to targeting dynamic PPI surfaces, optimizing drug-like properties, and validating clinical biomarkers, the therapeutic advantages of this strategy are significant. Hsp90–Cdc37 disruptors represent a promising frontier in precision oncology, offering a refined, selective, and less toxic approach to targeting cancer cell survival networks. Continued multidisciplinary research is expected to drive these agents toward successful clinical translation.

## Abstract 2

Disrupting the Hsp90–Cdc37 interaction offers a selective strategy to degrade oncogenic kinases, avoid heat shock responses, and enhance the efficacy of kinase-targeted cancer therapies.

## Graphical abstract

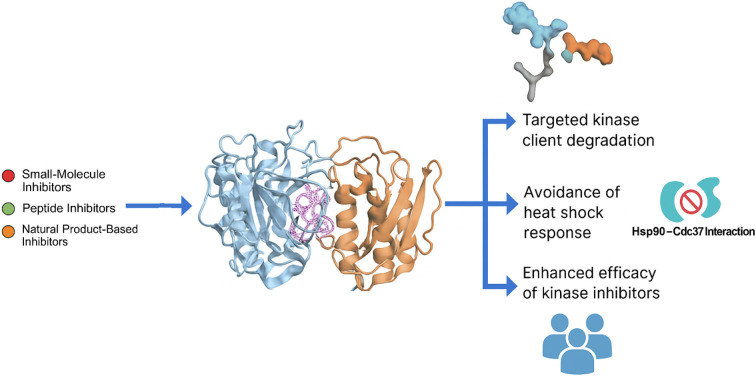

## 1. Introduction

Heat shock protein 90 (Hsp90) is an evolutionarily conserved molecular chaperone that governs the folding, maturation, and stabilization of a wide range of client proteins, including kinases, transcription factors, and hormone receptors.^1,2^ These client proteins are pivotal in cellular homeostasis, and many are directly involved in oncogenesis, tumor progression, and metastasis.^3,4^ Consequently, Hsp90 has long been recognized as a promising target in cancer therapeutics.^5,6^ Unlike general molecular chaperones, Hsp90 operates through a highly regulated ATP-dependent conformational cycle and relies on a network of co-chaperones to achieve its function, among which cell division cycle 37 (Cdc37) stands out as a critical mediator for the recruitment and stabilization of kinase clients.^7^

Hsp90 functions as a central hub in the proteostasis network, interacting with over 200 client proteins and numerous co-chaperones that regulate its activity, localization, and client specificity.^8^ These interacting partners include kinases (*e.g.*, Akt, CDK4, Raf-1), steroid hormone receptors (*e.g.*, glucocorticoid and androgen receptors), transcription factors (*e.g.*, HSF1, p53), and cochaperones such as Hop, p23, Aha1, and Cdc37.^9^ The diversity of these interactions reflects Hsp90's multifaceted roles in signaling, proliferation, and stress response. Among these, the interaction with Cdc37 is uniquely specialized for protein kinase clients, making it a particularly attractive and selective target for cancer therapy.^10^

The Hsp90–Cdc37 complex serves as a specialized machinery that facilitates the maturation of approximately 60% of all known kinases, including many oncogenic drivers such as Akt, Raf, CDK4, and HER2. Cdc37 not only acts as an adaptor that links kinases to Hsp90 but also modulates their conformation, protecting them from proteasomal degradation.^11–13^ This client-specific chaperoning function of the Hsp90–Cdc37 system positions it as a key node in cancer cell signaling networks, making it an attractive and selective therapeutic target.^12^ Traditional Hsp90 inhibitors, such as geldanamycin and its derivatives, have largely focused on blocking the ATPase activity at the N-terminal domain of Hsp90.^14,15^ While preclinical studies have demonstrated significant antitumor activity, clinical outcomes have been modest due to several inherent limitations.^14^ These include the induction of the heat shock response (HSR), a cytoprotective mechanism that leads to the upregulation of other heat shock proteins like Hsp70 and Hsp27, thereby blunting the therapeutic efficacy.^3,16^ Furthermore, broad inhibition of Hsp90 affects numerous client proteins across normal and malignant tissues, resulting in dose-limiting toxicities.^1,17^

**Fig. 1:**
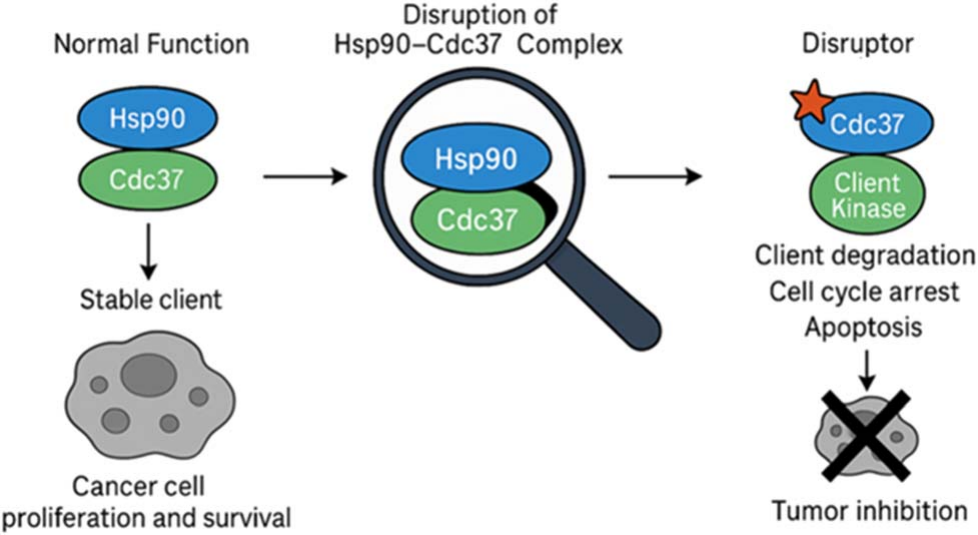
Schematic diagram for the disruption of Hsp90–Cdc37 complex as a strategy for targeted cancer therapy.

To circumvent these challenges, recent research has pivoted toward targeting specific protein–protein interactions (PPIs) within the Hsp90 chaperone machinery, particularly the interaction between Hsp90 and Cdc37 (Fig. 1).^13,18^ Disruption of the Hsp90–Cdc37 complex presents several advantages: it selectively impairs the maturation of kinase clients critical for tumor growth, minimizes the activation of compensatory stress pathways, and offers the potential for greater therapeutic specificity and reduced systemic toxicity.^19–21^ Notably, agents that disrupt this interaction—including natural products like celastrol and withaferin A, rationally designed small molecules, and engineered peptides—have shown potent antitumor activity in preclinical models without eliciting the canonical heat shock response.^22^ Moreover, insights from structural biology, molecular docking, and atomistic simulations have shed light on the key binding interfaces and dynamic hotspots within the Hsp90–Cdc37 complex, informing the rational design of new classes of inhibitors.^23,24^ Structure–activity relationship (SAR) studies have further refined lead compounds to enhance their potency, selectivity, and drug-like properties.^25,26^

In this review, we aim to provide a comprehensive and critical examination of the Hsp90–Cdc37 axis as a therapeutic target. We will explore the molecular basis of the Hsp90–Cdc37 interaction, discuss the various chemical and biological strategies employed to disrupt this complex, summarize the biological effects and therapeutic potential of these disruptors, and highlight the current challenges and future directions in the field. By focusing on the selective modulation of this key chaperone–co-chaperone interaction, we outline an emerging paradigm in the development of next-generation anticancer therapies.

## 2. Molecular basis of Hsp90–Cdc37 interaction

### 2.1 Structural insights into the Hsp90–Cdc37 interface

**Fig. 2:**
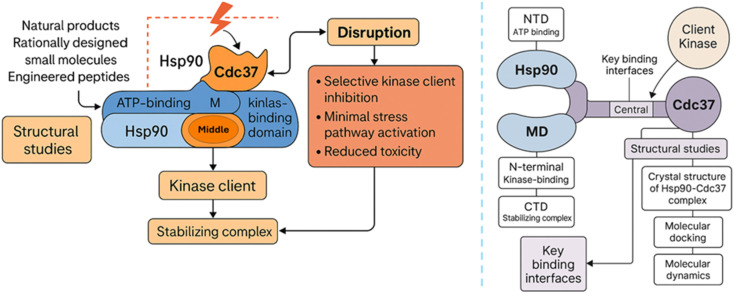
Structural insights into Hsp90–Cdc37 PPI disruption and its therapeutic implications.

The formation of the Hsp90–Cdc37 complex is a finely orchestrated event essential for the maturation of a broad range of protein kinases.^27,28^ Hsp90, a homodimeric molecular chaperone, consists of three domains: an N-terminal domain (NTD) responsible for ATP binding, a middle domain (MD) crucial for client binding and regulation, and a C-terminal domain (CTD) important for dimerization.^29,30^ Cdc37, on the other hand, acts as a kinase-specific adaptor and is structurally characterized by three regions: an N-terminal kinase-binding domain, a central domain that interacts with Hsp90, and a C-terminal domain that stabilizes the overall complex.^13,31,32^ Cryo-electron microscopy (cryo-EM) structures of the Hsp90–Cdc37–kinase complexes, such as the Hsp90–Cdc37–Cdk4 assembly, have provided significant insights into the nature of this interaction.^28,33^ Cdc37 bridges Hsp90 and client kinases by simultaneously engaging both, thereby facilitating their stabilization and maturation.^13,32,34^ Importantly, Cdc37 binding induces conformational rearrangements within Hsp90 that prime it for kinase loading, essentially “activating” the chaperone for selective client recruitment.^35^ The Hsp90–Cdc37 interaction primarily involves the N-terminal domain of Hsp90 and the middle domain of Cdc37, forming a highly specific protein–protein interface distinct from the ATP-binding pocket, making it an attractive target for selective therapeutic disruption.^36^ To visualize the structural organization of the Hsp90–Cdc37 complex and the mechanism by which its disruption confers selective antitumor effects, see Fig. 2. This model summarizes how structural and computational insights into the Hsp90–Cdc37 interface have enabled the rational development of novel PPI-disrupting agents with improved specificity and reduced toxicity profiles.

**Fig. 3:**
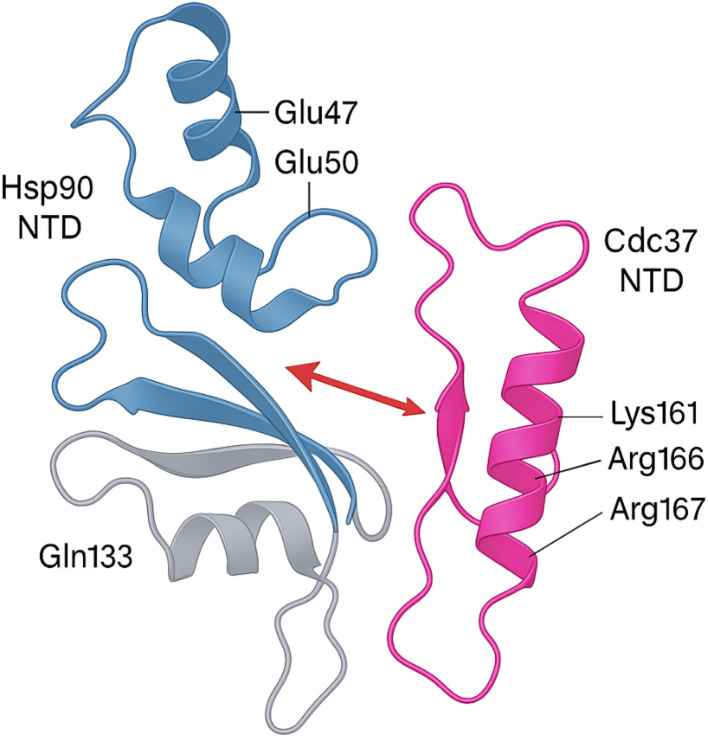
A ribbon representation of the asymmetric Hsp90–Cdc37 complex solved at 2.7 Å. The basic triad on the Cdc37 N-terminal domain (Lys161, Arg166, Arg167, magenta) clamps onto an acidic patch on the Hsp90 N-terminal domain (Glu47, Glu50, blue) while Gln133 (grey) anchors a hydrogen-bond relay across the hinge between the Hsp90 N- and middle domains. Mutating either Glu47/Glu50 or Arg166/Arg167 abolishes cochaperone binding and prevents kinase loading, underscoring these residues as the primary electrostatic hot-spot.

As illustrated in Fig. 3, the crystal structure of the human Hsp90 NTD/MD bound to the Cdc37 NTD (PDB 2CG9) reveals an electrostatic “clamp” in which the basic triad Lys161–Arg166–Arg167 of Cdc37 docks against an acidic patch formed by Glu47 and Glu50 on Hsp90, while Gln133 stabilises a hydrogen-bond relay across the NTD–MD hinge. Disruption of either side of this salt-bridge (*e.g.*, Glu47 → Ala or Arg166/Arg167 → Ala mutations) abolishes cochaperone binding and prevents client-kinase loading, underscoring these residues as druggable hot-spots for the small molecules, natural products and peptide mimetics detailed in Sections 3.

### 2.2 Key residues and domains involved

Molecular modeling, mutagenesis studies, and structural analyses have identified several key residues at the Hsp90–Cdc37 interface critical for complex formation.^29,34,36^ On Hsp90, residues within the N-terminal domain, particularly Glu33 and Glu47, are pivotal contact points that anchor Cdc37 binding.^37,38^ Cdc37's binding involves a central stretch of residues, including a highly conserved arginine residue (Arg167) that engages in strong electrostatic interactions with Hsp90.^32,38^ Additional hydrophobic and polar interactions between specific residues contribute to the stability and specificity of the complex.^34,38,39^ The interface forms a relatively shallow and extended binding surface, characteristic of many transient PPIs but with hotspots that are amenable to small molecules or peptide targeting.^13,18,23^ The recognition of this interface has enabled the rational design of disruptors, such as celastrol derivatives and peptides derived from the Cdc37 sequence, which competitively bind and interfere with complex formation without affecting the global ATPase activity of Hsp90.^1,18,40^

### 2.3 Role of Cdc37 as a kinase-specific co-chaperone

**Fig. 4:**
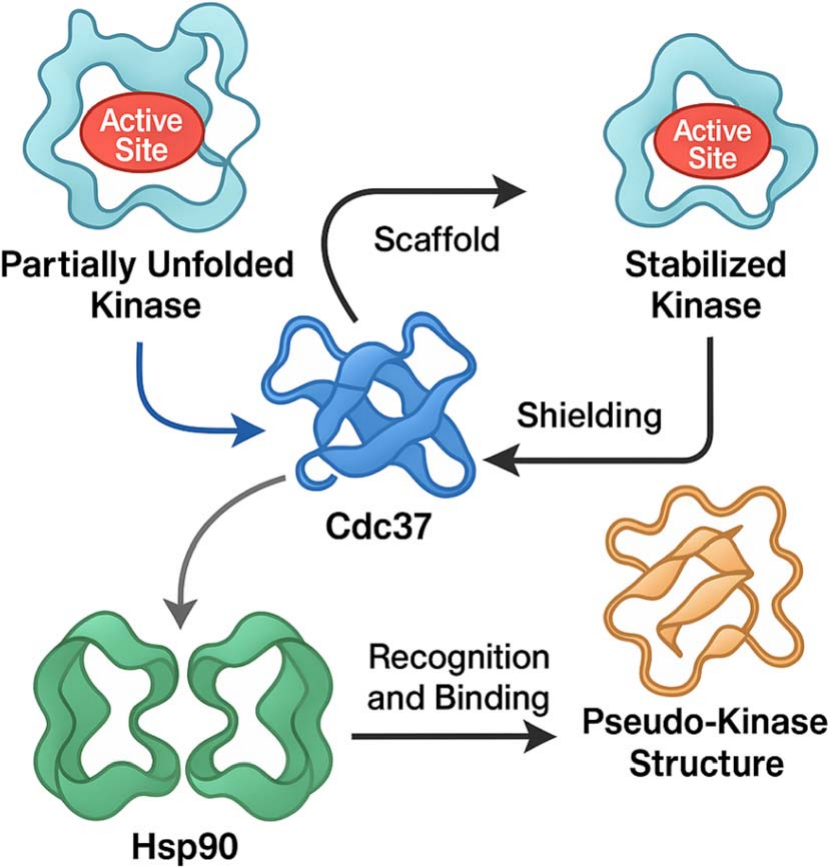
Molecular mechanism of Cdc37 as a kinase-specific co-chaperone in complex with Hsp90.

Cdc37 serves as a specialized co-chaperone that primarily targets protein kinases, a unique subset of Hsp90 clients characterized by their conformational instability and stringent chaperone dependence.^32,41^ Functionally, Cdc37 performs two main roles: it acts as a scaffold to deliver partially unfolded kinase clients to Hsp90, and it stabilizes these clients by shielding their labile active sites during early stages of folding.^13,18^ Importantly, Cdc37 itself exhibits a pseudo-kinase structure, allowing it to mimic substrate kinases and thus efficiently recognize and bind to the kinase domain of client proteins.^29^ This selective recruitment mechanism ensures that kinases are preferentially stabilized by Hsp90, highlighting the centrality of the Hsp90–Cdc37 axis in maintaining oncogenic signaling networks.^13,32^ Given its essentiality and specificity, targeting Cdc37–Hsp90 interaction offers a unique therapeutic window to selectively degrade oncogenic kinases without broadly affecting the entire proteome, thereby minimizing off-target effects associated with conventional Hsp90 ATPase inhibitors.^42^ This kinase-specific chaperoning mechanism is schematically illustrated in Fig. 4, which depicts how Cdc37 recruits and stabilizes kinase clients through its pseudo-kinase structure in cooperation with Hsp90.

## 3. Strategies for disrupting the Hsp90–Cdc37 complex

The selective disruption of the Hsp90–Cdc37 PPI has emerged as a powerful therapeutic strategy in oncology.^13,23^ Unlike classical ATPase inhibitors of Hsp90, PPI disruptors aim to selectively degrade kinase clients without broadly affecting the global chaperone function, thus offering greater specificity and reduced systemic toxicity.^17,43^ Strategies explored to date include small molecule inhibitors, peptides and peptidomimetics, and natural products or their derivatives (Table 1).^13,17^

**Table 1:** Reported Hsp90–Cdc37 disruptors and their potency

Compound/peptide	Mechanism	Potency (units & context)	Cancer model/system	Key notes
Platycodin D	Disrupts PPI	Not determined	NSCLC (combo with everolimus)	No Hsp70 induction; feedback termination
Celastrol-triazole (6)	Disrupts PPI	IC_50_ (cell-based, MDA-MB-231) = 0.34 μM	Breast	Covalent thiol binding
Compound 10	Disrupts PPI	IC_50_ (cell-based) = 27 μM; *K*_D_ (biochemical) = 40 μM	MCF-7, SKBR3, A549	Moderate affinity
DDO-5994	Hydrophobic pocket	IC_50_ (biochemical) = 6.34 μM; *K*_D_ = 5.52 μM	*In vitro*	Targets hydrophobic core (Phe213)
DCZ3112	Hsp90 NTD binder	IC_50_ (cell-based, SK-BR-3) = 7.9 μM; IC_50_ (cell-based, BT-474) = 4.6 μM	HER2^+^ breast	Overcomes trastuzumab resistance
Compound 11g	Disrupts PPI	IC_50_ (cell-based, A549) = 0.14 μM	A549	Migration inhibition; apoptosis
DDO-5936	Glu47 Hsp90 binder	IC_50_ (cell-based, HCT116) = 8.99 ± 1.21 μM	HCT116	CDK4 degradation
K-Ras-selective molecules	Hsp90–Cdc37 disruption	IC_50_ (biochemical) = 4–44 μM	K-Ras-driven tumours	No heat-shock response
Compound 8c	Hsp90 binder	*K*_D_ = 70.8 μM; IC_50_ (MCF-7) = 20.0 ± 1.2 μM; IC_50_ (SK-N-MC) = 12.8 ± 0.9 μM; IC_50_ (THP-1) = 33.9 ± 8.5 μM	MCF-7, SK-N-MC, THP-1	Apoptosis induction
Compound 13g	Hsp90 binder	*K*_D_ = 73.3 μM; IC_50_ (MCF-7) = 19.3 ± 2.0 μM; IC_50_ (SK-N-MC) = 20.0 ± 1.5 μM; IC_50_ (THP-1) = 41.5 ± 6.3 μM	MCF-7, SK-N-MC, THP-1	Apoptosis induction
Pep-5	Peptide disruptor	*K*_D_ (biochemical) = 5.99 μM	—	Smallest stable peptide
KTGDEK peptide	Peptide inhibitor	*K*_D_ (biochemical) = 2.78 μM	HCC	Pre-cyclic peptidomimetic
TAT-DDO-59120	Peptidomimetic	IC_50_ (cell-based, HCT116) = 12.82 μM	HCT116	Enhanced cell penetration
Celastrol	Cdc37 binder	Active 1–5 μM (cell-based)	Panc-1	ATP-independent; metastasis suppression
Withaferin A	Interface disruptor	*In silico* (no IC_50_ reported)	Various	Thermodynamic stabilization
Okicamelliaside	N-Terminal Hsp90 binder	*K*_D_ = 6.45 μM; IC_50_ (cell-based, A549) ≈ 22.7 μM	A549	Targets Glu47

### 3.1 Natural products and semi-synthetic derivatives

#### 3.1.1 Platycodin D

**Fig. 5:**
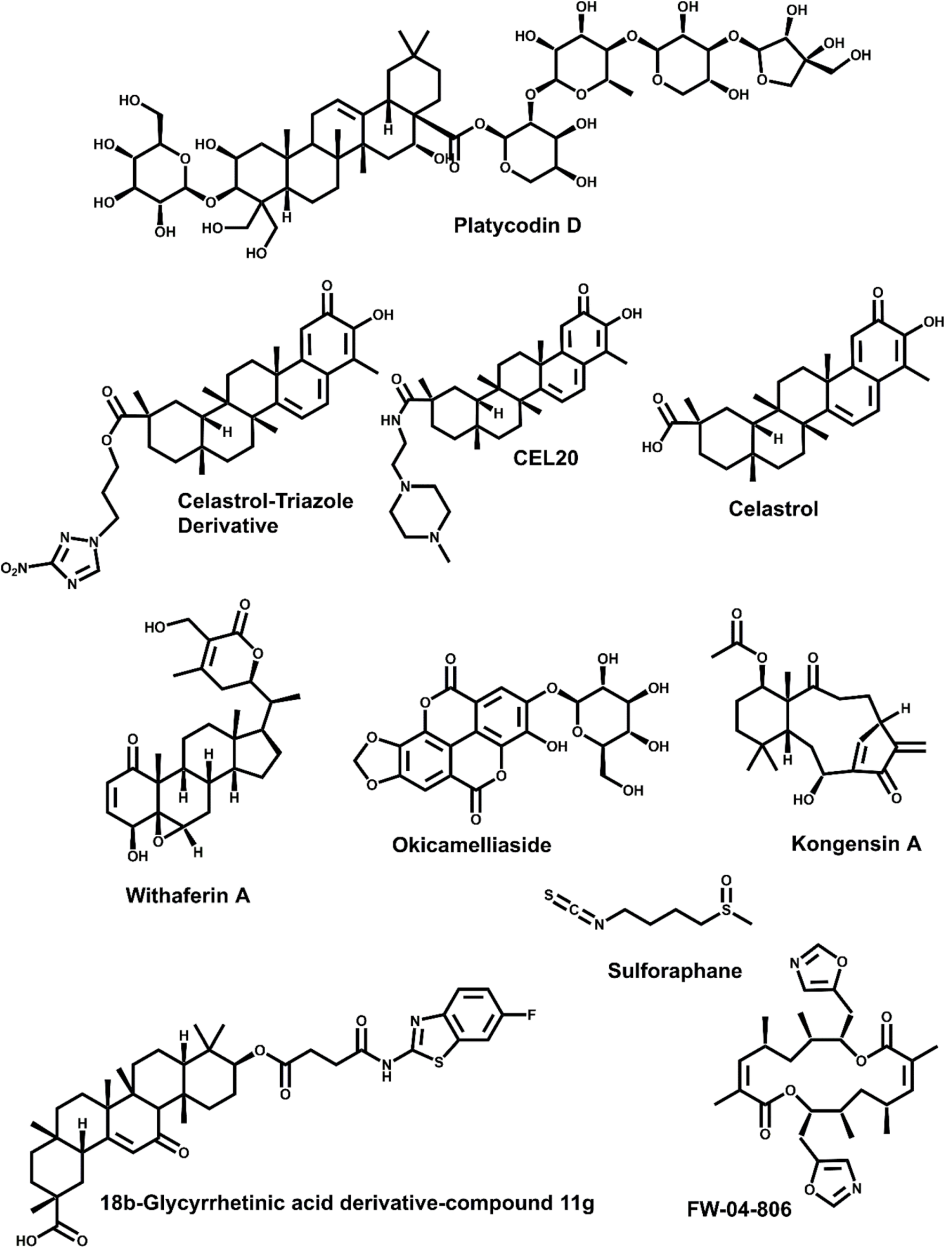
Structures of Hsp90–Cdc37 natural inhibitors.

A novel saponin isolated from *Platycodonis Radix* (Fig. 5), shown to disrupt Hsp90–Cdc37 interaction without affecting ATPase activity. PD led to degradation of multiple client proteins and blocked feedback survival signals induced by mTOR inhibition in non-small cell lung cancer cells. When combined with Everolimus (mTOR inhibitor), PD enhanced antiproliferative and apoptotic effects by suppressing EGFR and IGF1R expression and AKT signaling.^44^

#### 3.1.2 Celastrol

Celastrol, a natural quinone methide triterpenoid, has emerged as a unique modulator of the Hsp90–Cdc37 chaperone complex through a thiol-reactive, allosteric mechanism (Fig. 5).^45^ Unlike classical Hsp90 inhibitors that target the ATP-binding site, celastrol binds preferentially to Hsp90 and interferes with its interaction with the cochaperone Cdc37.^45^ Notably, it does not inhibit ATP binding to Hsp90, allowing the chaperone's catalytic cycle to proceed until its disruption at the cochaperone interface. Mechanistically, celastrol's inhibitory action has been attributed to its ability to covalently modify thiol groups and alter the conformation of the Hsp90 C-terminal domain.

At pharmacologically relevant concentrations (1–5 μM), celastrol induced 70–80% degradation of Hsp90 client proteins such as Cdk4 and Akt and triggered a robust 12-fold increase in Hsp70 expression in Panc-1 pancreatic cancer cells.^46^*In vivo*, administration of celastrol at 3 mg kg^−1^ resulted in more than 80% suppression of tumor metastasis, highlighting its therapeutic efficacy in aggressive cancer models.^46,47^

Zhang *et al.* (2009) provided detailed biochemical evidence for celastrol's mechanism of action using a reconstituted protein system.^45^ Through ELISA and GST pull-down assays, they showed that celastrol disrupts the Hsp90–Cdc37 complex by binding to the C-terminal region of Hsp90—distinct from the ATP pocket—and inhibiting its ATPase activity. Proteolytic fingerprinting further confirmed that celastrol induces conformational changes within the chaperone, supporting an allosteric inhibition model. These findings underscore the potential of C-terminal-targeting agents to modulate chaperone function selectively, without interfering with nucleotide binding.^45^

Expanding on this mechanistic insight, Zhang *et al.* demonstrated celastrol's potent antitumor activity in pancreatic cancer models.^47^ Molecular docking and dynamic simulations revealed that celastrol disrupts the critical Glu33–Arg167 interface at the Hsp90–Cdc37 junction. Experimental validation confirmed that celastrol (10 μM) effectively dissociates this complex in Panc-1 cells. Functionally, this disruption led to client protein degradation, apoptosis induction, and marked tumor regression in xenograft and transgenic mouse models. With an *in vitro* IC_50_ of 3 μM—significantly lower than that of geldanamycin—celastrol exemplifies a new class of non-ATP-competitive Hsp90 inhibitors that achieve targeted disruption of cochaperone-mediated oncogenic signaling.^47^

#### 3.1.3 Celastrol-triazole derivative

Li *et al.* designed 27 celastrol-triazole derivatives (Fig. 5) to enhance disruption of the Hsp90–Cdc37 PPI and improve anticancer efficacy.^48^ Among these, compound 6 showed the strongest antiproliferative activity, with an IC_50_ of 0.34 ± 0.01 μM in MDA-MB-231 cells—approximately 3.5-fold more potent than celastrol itself. Mechanistic studies confirmed that compound 6 disrupted the Hsp90–Cdc37 complex more effectively than CEL, downregulated client proteins Cdk4 and p-Akt, induced G0/G1 cell cycle arrest, and triggered dose-dependent apoptosis.^48^ The compound also exhibited increased covalent binding to thiols, suggesting enhanced target engagement *via* its modified quinone methide structure. These findings highlight compound 6 as a promising lead for the development of targeted Hsp90–Cdc37 PPI inhibitors in oncology.^48^

#### 3.1.4 Withaferin A

Grover *et al.* employed a computational approach to investigate the potential of Withaferin A (WA), a withanolide derived from *Withania somnifera*, to disrupt the Hsp90–Cdc37 chaperone–cochaperone complex (Fig. 5).^49^ Flexible and semi-flexible molecular docking studies revealed that WA binds preferentially at the Hsp90–Cdc37 interface, specifically displacing key hydrogen bonds involving Gln133 (Hsp90) and Arg166/Arg167 (Cdc37) that are essential for complex stability. The binding energy of WA to the active complex was −13.95 kcal mol^−1^, significantly stronger than its binding to Hsp90 alone (−9.10 kcal mol^−1^), suggesting selective affinity for the assembled interface. Molecular dynamics simulations confirmed the thermodynamic stability of the WA-bound complex and showed that WA effectively disrupts inter-chain interactions, leading to destabilization of the chaperone assembly.^49^ These findings support WA as a promising scaffold for selective inhibition of the Hsp90–Cdc37 interaction, offering an alternative to traditional ATP-binding Hsp90 inhibitors with potentially improved specificity.^49^

#### 3.1.5 Okicamelliaside

Cheng *et al.* identified okicamelliaside (Fig. 5), an ellagic acid derivative from *Camellia nitidissima*, as a novel small-molecule disruptor of the Hsp90–Cdc37 PPI that selectively targets the N-terminal chaperone pocket of Hsp90.^37^ Using a combination of target fishing, molecular docking, and dynamic simulations, the study demonstrated that OCS binds competitively at the Glu-47 site—a key residue in the Hsp90–Cdc37 interface—with a dissociation constant (*K*_D_ = 6.45 μM).^37^ OCS disrupted the PPI without interfering with Hsp90's ATPase activity or affecting non-kinase client proteins, unlike classic ATP-competitive inhibitors. Functionally, OCS selectively downregulated phosphorylation of kinase clients (*e.g.*, CDK4, P-AKT_473_, P-ERK1/2) and induced apoptosis in A549 lung cancer cells (IC_50_ ≈ 22.7 μM). *In vivo*, OCS significantly reduced tumor growth and disrupted Hsp90–Cdc37 co-localization in xenografts with minimal toxicity.^37^ These findings establish OCS as a promising lead compound for selectively modulating chaperone–cochaperone interactions in cancer therapy.

#### 3.1.6 18β-Glycyrrhetinic acid derivative (compound 11g)

Compound 11g demonstrated a low micromolar IC_50_ in assays targeting Hsp90–Cdc37 disruption (Fig. 5). It significantly inhibited A549 lung cancer cell migration and proliferation, induced apoptosis, and arrested cells at S phase.^50^ Compound 11g, featuring a 5-fluoro (5-F) substituent, demonstrated potent inhibition of the Hsp90-Cdc37 interaction with a submicromolar IC_50_ of 0.14 μM. Positional analysis of substituents on the benzothiazole ring revealed that modifications at the C-5 position generally conferred greater activity than those at C-7. Accordingly, 11g emerged as the most effective inhibitor among the tested analogs.^50^ The incorporation of a benzothiazole ring in 11g is consistent with the broader pharmacological relevance of 2-aminobenzothiazole scaffolds, which have recently been reviewed as privileged anticancer chemotypes—including several molecules that modulate the Hsp90–Cdc37 interface.^51^

#### 3.1.7 Other natural products inhibitors

In addition to the ligands already reviewed, several naturally derived small molecules have been validated as Hsp90–Cdc37 disruptors and merit inclusion (Fig. 5). FW-04-806, a macrolide–resorcylic-acid lactone isolated from *Streptomyces* sp., binds the N-terminal domain of Hsp90, prevents cochaperone docking, and triggers client-kinase degradation, yielding potent antiproliferative effects in HER2-overexpressing breast-cancer models.^52^ Sulforaphane, the isothiocyanate abundant in cruciferous vegetables, similarly docks at the Hsp90–p50Cdc37 interface; immunoprecipitation and molecular-modelling studies show complex destabilization accompanied by marked suppression of pancreatic-cancer growth *in vitro* and in orthotopic models.^53^ Kongensin A, an *ent*-kaurane diterpenoid from *Isodon* spp., covalently modifies Cys420 in the Hsp90 middle domain, ejects Cdc37, and induces apoptosis across a spectrum of tumor lines, exemplifying a non-canonical allosteric mechanism.^18^ These agents further broaden the pharmacological repertoire for selectively modulating the Hsp90–Cdc37 axis.

### 3.2 Synthetic small-molecule inhibitors

#### 3.2.1 DDO-5936

**Fig. 6:**
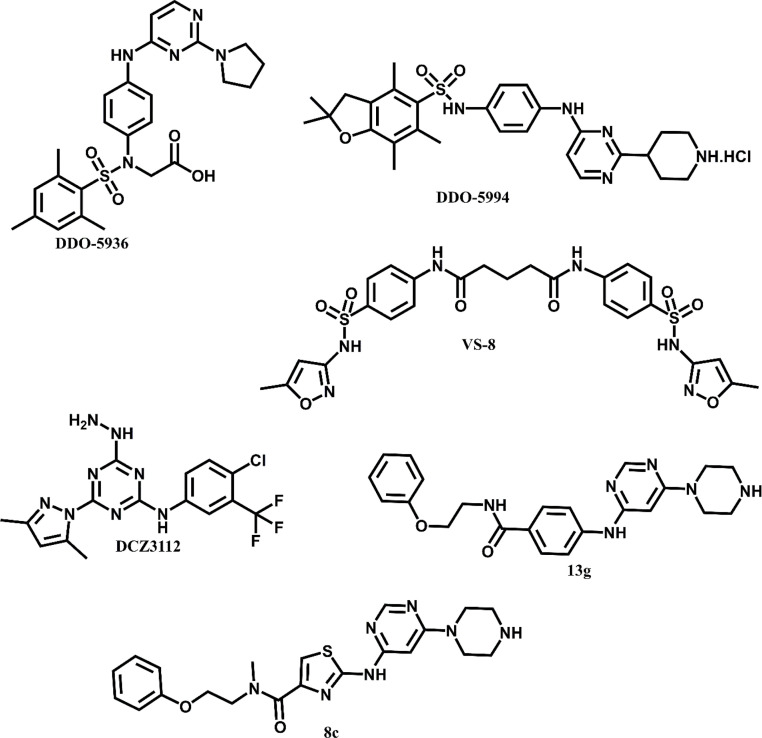
Structures of Hsp90–Cdc37 synthetic inhibitors.

Wang *et al.* identified DDO-5936 (Fig. 6) as the first small-molecule inhibitor to selectively disrupt the Hsp90–Cdc37 PPI *via* binding to a previously unrecognized site on Hsp90 involving Glu47 and Gln133.^54^ Unlike classical ATPase inhibitors, DDO-5936 displayed minimal impact on Hsp90 ATPase activity (IC_50_ > 100 μM) and selectively degraded kinase clients such as CDK4 and CDK6 without inducing a heat shock response. In HCT116 colorectal cancer cells, it showed potent antiproliferative effects with an IC_50_ of 8.99 ± 1.21 μM, and efficacy was confirmed in a xenograft model, demonstrating a correlation between Hsp90–Cdc37 expression and sensitivity to the compound.^54^ These findings position DDO-5936 as a promising lead for developing functionally specific Hsp90 modulators.^54^

#### 3.2.2 DDO-5994

Zhang *et al.* identified a novel hydrophobic pocket centered on Phe213 in Hsp90 and designed a series of inhibitors to exploit this site for selectively disrupting the Hsp90–Cdc37 PPI.^55^ Their optimized compound, DDO-5994 (Fig. 6), exhibited significantly enhanced binding affinity (*K*_D_ = 5.52 μM) and antiproliferative activity (IC_50_ = 6.34 μM) compared to the parent compound 11 (*K*_D_ > 20 μM, IC_50_ > 50 μM).^55^ DDO-5994 directly bound Hsp90, disrupted the Hsp90–Cdc37 complex in a dose-dependent manner, induced CDK4/6 degradation, G0/G1 cell cycle arrest, and showed *in vivo* antitumor efficacy in HCT116 xenografts without triggering a heat shock response. This study highlights the Phe213 pocket as a druggable site for future design of selective PPI inhibitors targeting the Hsp90–Cdc37 axis.^55^

#### 3.2.3 Structure-guided VS-8 derivative 10

Wang *et al.* applied a structure-based virtual screening strategy to identify small-molecule disruptors of the Hsp90–Cdc37 PPI, leading to the discovery of compound 10.^56^ This compound exhibited improved potency compared to its parent hit VS-8 (Fig. 6), with an IC_50_ of 27 μM and a *K*_D_ of 40 μM, alongside antiproliferative effects in MCF-7, SKBR-3, and A549 cancer cell lines (IC_50_ = 26, 15, and 38 μM, respectively).^56^ Mechanistic studies confirmed that compound 10 disrupted the Hsp90–Cdc37 complex in a concentration-dependent manner, downregulated client proteins Akt and Cdk4, and did not interfere with ATP binding. This work highlights compound 10 as a promising non-natural scaffold for the development of selective Hsp90–Cdc37 inhibitors.^56^

#### 3.2.4 DCZ3112

DCZ3112 binds to the N-terminal domain of Hsp90 and selectively disrupts the Hsp90–Cdc37 interaction without inhibiting ATPase activity. DCZ3112 inhibited proliferation of HER2-positive breast cancer cells and overcame trastuzumab resistance, while reducing AKT and ERK phosphorylation (Fig. 6).^57^ DCZ3112 selectively suppressed the growth of HER2-positive breast-cancer cells, yielding IC_50_ values of 7.9 μM in SK-BR-3 and 4.6 μM in BT-474. In contrast, HER2-negative cell lines were largely unaffected (IC_50_ > 25 μM), with the lone exception of MDA-MB-468, which displayed greater sensitivity.^57^

#### 3.2.5 K-Ras-selective Hsp90–Cdc37 interface inhibitors

An ultra-large *in silico* screen of >7 million drug-like molecules yielded four benzyl-pyrimidinone scaffolds—FAP-260, FAP-881, FAP-967 and FAP-1063—that bind a shallow pocket surrounding Glu47 in the N-terminal domain of Hsp90 (*K*_D_ = 5.1–11 μM) and prevent Cdc37 docking. These compounds degraded the Hsp90 client HIF-1α, suppressed galectin-3, dismantled K-Ras nanoclusters, and selectively inhibited the proliferation of KRAS-mutant MIA-PaCa-2 and A549 cells (IC_50_ = 4–18 μM) without triggering a heat-shock response; the lead analogue FAP-967 reduced tumor volume by 62% in MIA-PaCa-2 xenografts at 50 mg kg^−1^.^58^ Complementing these synthetics, the macrodiolide elaiophylin was shown to covalently disrupt the same Hsp90–Cdc37 interface, likewise depleting HIF-1α and galectin-3 and inducing K-Ras-selective apoptosis in cancer cells, thereby reinforcing the nanocluster-dependent mechanism of KRAS selectivity.^59^

#### 3.2.6 Compounds 8c and 13g

Ligand-based screening yielded these two compounds with binding affinities *K*_d_ = 70.8 μM and 73.3 μM, respectively (Fig. 6). Both reduced Hsp90 client proteins in MCF-7 cells and induced apoptosis in SK-N-MC Ewing sarcoma cells.^36^ Compounds 8c and 13g emerged as the most promising Hsp90–Cdc37 PPI inhibitors. Compound 8c demonstrated potent antiproliferative activity with IC_50_ values of 20.0 ± 1.2 μM in MCF-7 breast cancer cells, 12.8 ± 0.9 μM in SK-N-MC Ewing sarcoma cells, and 33.9 ± 8.5 μM in THP-1 leukemia cells. Similarly, compound 13g exhibited IC_50_ values of 19.3 ± 2.0 μM, 20.0 ± 1.5 μM, and 41.5 ± 6.3 μM in the respective cell lines. Both compounds disrupted the Hsp90–Cdc37 interaction and reduced levels of key Hsp90 client proteins without triggering a heat shock response, supporting their potential as targeted anticancer agents.^36^

### 3.3 Peptides and peptidomimetics

Peptide-based strategies offer high specificity for the Hsp90–Cdc37 interface, mimicking critical binding motifs:

#### 3.3.1 Pep-1 and Pep-5

In an effort to develop selective modulators of the Hsp90–Cdc37 PPI, Wang *et al.* designed and optimized a series of Cdc37-derived peptides.^60,61^ Through molecular docking and MD simulations, they identified Pep-5 as the shortest and most potent candidate, exhibiting a K_d_ of 5.99 μM and improved ligand efficiency (BEI = 6.21) compared to the parent peptide Pep-1 (*K*_D_ = 6.90 μM; BEI = 3.18). Pep-5 maintained high binding stability and effectively disrupted the Hsp90–Cdc37 complex *in vitro*, as demonstrated by GST pull-down assays. Notably, it did not interfere with ATP binding in a fluorescence polarization assay, indicating selective targeting of the PPI interface rather than the Hsp90 ATP-binding site.^60,61^ These findings provide a promising foundation for the development of small-molecule Hsp90–Cdc37 PPI inhibitors.^60,61^

#### 3.3.2 KTGDEK-derived peptides

**Fig. 7:**
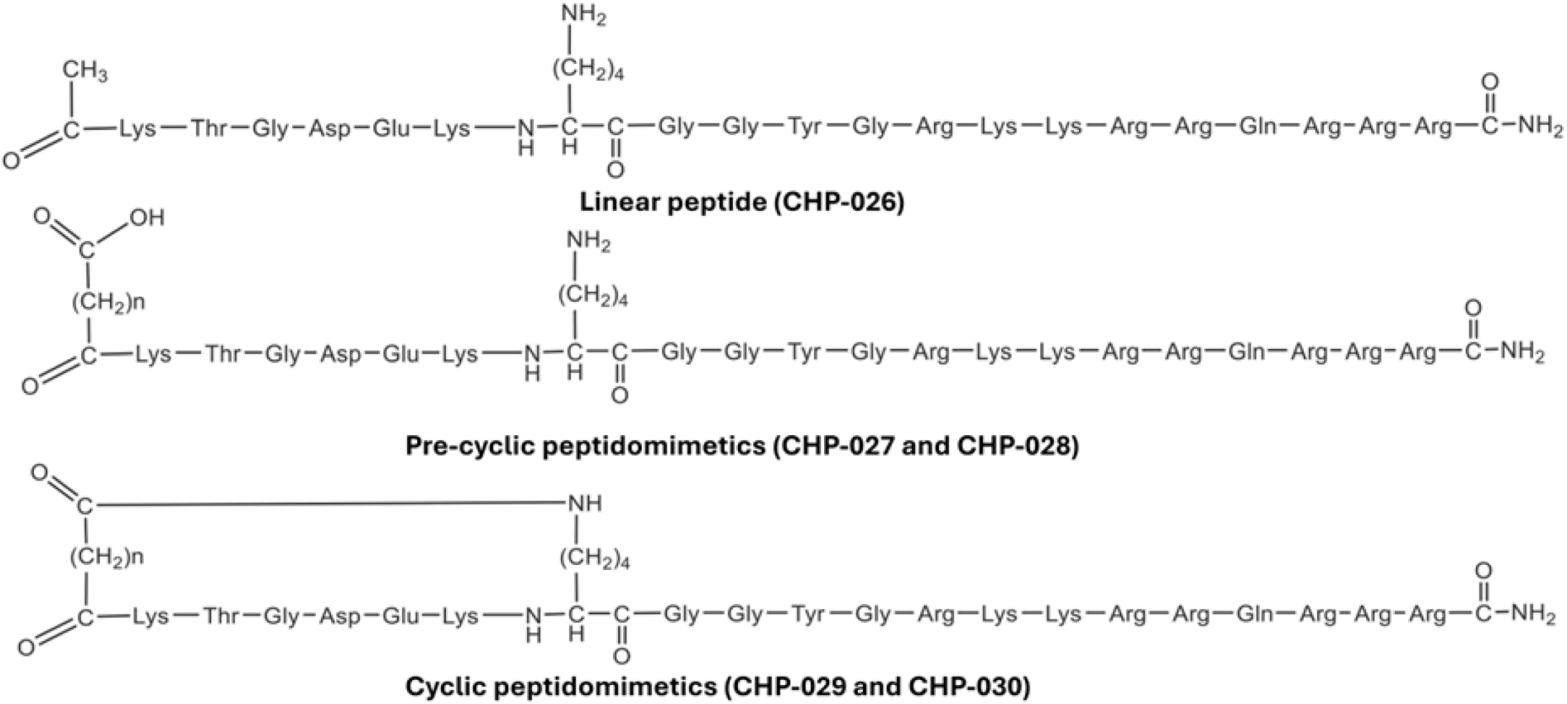
Structures of Hsp90–Cdc37 peptide inhibitors.

Sukumaran *et al.* introduced a rational design strategy to develop peptide-based inhibitors targeting the Hsp90–Cdc37 interaction as a therapeutic avenue in hepatocellular carcinoma.^62^ A linear hexapeptide derived from a conserved KTGDEK motif was synthesized and modified into a library of pre-cyclic and cyclic peptidomimetics (Fig. 7). Among these, the pre-cyclic derivative CHP-028 demonstrated the most favorable properties, with a binding affinity (*K*_D_) of 16.4 μM, and effectively competed with Cdc37 for binding to Hsp90 (competitive *K*_D_ = 2.78 μM). Functionally, CHP-028 significantly inhibited HCC cell proliferation and migration, elevated caspase-3 levels, and downregulated phosphorylated MEK1/2, without affecting total MEK1/2 levels. These findings validate a structured peptide-engineering approach for selectively modulating Hsp90–Cdc37 PPIs in cancer.^62^

#### 3.3.3 TAT-DDO-59120

Using a kinase pseudosubstrate-based approach, researchers identified TAT-DDO-59120 as a selective peptide inhibitor capable of disrupting the HSP90–CDC37 PPI.^63^ The peptide was shown to bind directly to HSP90 both intra- and extracellularly, effectively impairing the PPI and demonstrating antiproliferative activity in HCT116 colorectal cancer cells (IC_50_ = 12.82 μM). This targeted strategy offers a rapid and rational alternative to conventional high-throughput screening for PPI inhibitors.^63^

#### 3.3.4 Structure-guided designed peptide inhibitors

D'Annessa *et al.* applied a structure- and dynamics-based peptide design approach to develop inhibitors targeting the Hsp90–Cdc37 interface.^64^ Utilizing bioinformatics tools such as PepCrawler and Pep-Whisperer, they identified short peptides—specifically Cdc37p3 and Cdc37p5—derived from the Cdc37 sequence that exhibited strong binding affinity to Hsp90.^64^ Molecular dynamics simulations confirmed that these peptides could stably associate with the Hsp90 binding interface and effectively disrupt the Hsp90–Cdc37 complex. Functional assays demonstrated that Cdc37p3 and Cdc37p5 inhibited the maturation of Cdk4, a kinase client dependent on Cdc37, without affecting the stability of non-Cdc37-dependent clients.^64^ Although these peptides lacked substantial cytotoxicity in cell-based assays, their strong mechanistic activity supports their potential as molecular tools and starting points for further optimization into drug-like peptidomimetics.^64^ This rational peptide engineering strategy highlights the feasibility of targeting transient and shallow protein–protein interaction surfaces using short, conformationally stabilized sequences.

## 4. Discovery and development of Hsp90–Cdc37 disruptors

The pursuit of selective Hsp90–Cdc37 disruptors has required innovative discovery strategies and sophisticated development techniques.^5,36^ Compared to classical Hsp90 ATPase inhibitors, disruptors of the Hsp90–Cdc37 interface face the inherent challenge of targeting relatively shallow and dynamic PPI surfaces.^13,18,23^ To overcome these barriers, a combination of structure-based design, virtual screening, natural product exploration, and rational peptide engineering has been utilized.^62,63^

### 4.1 Structure-based virtual screening and optimization

The emergence of high-resolution structural data, particularly the cryo-EM structure of the Hsp90–Cdc37–Cdk4 complex, has been instrumental in mapping key interaction hotspots such as Glu33 and Glu47 on Hsp90 and Arg167 on Cdc37.^33^ These structural insights have provided a robust framework for structure-based virtual screening (SBVS) efforts aimed at identifying the first generation of small-molecule disruptors targeting the Hsp90–Cdc37 interface.^65^ One early success was the identification of VS-8, which through iterative optimization yielded compound 10, demonstrating improved potency (IC_50_ = 27 μM) and binding affinity (*K*_D_ = 40 μM).^56^ Expanding the scope of screening, an ultra-large virtual screen encompassing over seven million compounds uncovered additional inhibitors with selective activity toward the K-Ras signaling axis, exhibiting inhibitory potencies ranging from 4 to 44 μM and effective suppression of both 2D cell proliferation and 3D tumor spheroid growth.^58^ In parallel, rational design approaches led to the discovery of DDO-5936, a compound specifically targeting the previously underexplored Glu47 residue on Hsp90.^54^ DDO-5936 effectively disrupted the Hsp90–Cdc37 interaction and showed promising antiproliferative activity in HCT116 colorectal cancer cells, further validating Glu47 as a druggable site for selective PPI modulation.^54^

### 4.2 Rational design based on hydrophobic core targeting

Further elucidation of the Hsp90–Cdc37 interaction interface has uncovered a previously underappreciated hydrophobic pocket centered on Phe213 in Hsp90, which plays a key role in stabilizing the chaperone–cochaperone complex.^5,34^ This discovery prompted efforts to exploit this pocket as a novel target site for small-molecule disruption of the PPI. Through strategic hydrophobic substitution focused on this region, researchers developed DDO-5994, a potent inhibitor that demonstrated a marked improvement in both binding affinity (*K*_D_ = 5.52 μM) and antiproliferative efficacy (IC_50_ = 6.34 μM).^55^ The success of DDO-5994 underscores the broader therapeutic potential of targeting discrete, hydrophobic microdomains within protein interfaces, providing a compelling strategy for the rational design of selective PPI inhibitors.^13,23^

### 4.3 *In silico* approaches and molecular dynamics simulations

Computational approaches have played a central role in advancing the discovery and optimization of enzyme and protein inhibitors.^66–68^ Atomistic simulations, including molecular dynamics and rigidity decomposition analyses, have enabled precise mapping of conformational flexibility within the Hsp90–Cdc37–Cdk4 complex, revealing instability hotspots at the client interface that may serve as vulnerable targets for disruption.^69^ Complementing these structural insights, binding free energy calculations and molecular docking have been instrumental in evaluating the thermodynamic stability of ligand-bound complexes, aiding in the rational prioritization of lead compounds based on predicted binding strength and specificity.^23^ Beyond static interactions, network modeling and analysis of allosteric regulation have uncovered critical intermolecular communication pathways and post-translational modification nodes that control client recruitment. These insights offer a systems-level understanding of how selective interference at key structural and regulatory sites can dismantle oncogenic chaperone signaling with high specificity.^10^

### 4.4 Structural characterization of inhibitor binding sites

Several Hsp90–Cdc37 disruptors have been characterized through crystallographic or cryo-EM studies, offering detailed insight into their binding mechanisms.^34^ For example, DDO-5936 has been shown *via* co-crystallization and molecular docking to bind a unique surface pocket near Glu47 and Gln133 on Hsp90, distinct from the ATP-binding site.^54^ Similarly, the celastrol–Hsp90 interaction has been modeled using biochemical reconstitution assays and docking simulations, indicating covalent binding to thiol groups in the C-terminal domain, which induces allosteric disruption of the Cdc37 interface.^70^ Okicamelliaside, identified by Cheng *et al.*, was shown through molecular docking and dynamics simulations to bind competitively at Glu47, a critical contact residue at the Hsp90–Cdc37 junction.^37^ For peptides, Pep-5 and CHP-028 have been structurally optimized through MD simulations and show stable interaction with key Cdc37 residues involved in Hsp90 binding, although crystal structures are not yet available. While crystal or cryo-EM structures of full ligand-bound Hsp90–Cdc37–inhibitor complexes remain limited, ongoing modeling and structure–activity relationship (SAR) studies have enabled increasingly precise predictions of binding modes.^33^

## 5. Mechanisms of action and biological effects

Disrupting the Hsp90–Cdc37 complex exerts profound biological effects distinct from conventional Hsp90 ATPase inhibition. Targeting this specific PPI selectively destabilizes kinase clients critical for oncogenic signaling, leading to impaired tumor cell survival with minimal activation of cytoprotective stress responses. A comprehensive understanding of the mechanisms underlying the action of Hsp90–Cdc37 disruptors is critical to appreciating their therapeutic potential.

### 5.1 Disruption of client protein maturation

The Hsp90–Cdc37 complex plays a critical role in the folding, stabilization, and activation of a wide array of protein kinases, many of which are central to oncogenic signaling. Disruption of this interaction prevents client kinases from achieving their mature conformation, rendering them unstable and leading to their rapid ubiquitination and subsequent degradation *via* the proteasome.^71,72^ This effect is particularly pronounced in key oncogenic kinases such as Akt, Raf-1, Cdk4, HER2, and EGFR, which are highly dependent on the chaperoning activity of the Hsp90–Cdc37 complex.^71,72^ For instance, celastrol effectively disrupted the Hsp90–Cdc37 interaction and induced 70–80% degradation of Cdk4 and Akt in Panc-1 pancreatic cancer cells.^47^ Similarly, FW-04-806 promoted degradation of HER2, Raf-1, and Akt in HER2-overexpressing SKBR3 cells, while platycodin D significantly reduced EGFR and IGF1R levels, particularly in combination with mTOR inhibitors.^73^ Additionally, rationally designed inhibitors such as DDO-5994 and DDO-5936 led to the downregulation of Cdk4 and other kinase clients of Hsp90.^54,55^ This selective destabilization of oncogenic kinases offers a strategic advantage over traditional ATP-competitive Hsp90 inhibitors, as it allows for targeted disruption of disease–relevant pathways without broadly impairing the proteome or triggering a global heat shock response.

### 5.2 Inhibition of key oncogenic signaling pathways

As kinase clients of the Hsp90–Cdc37 complex are destabilized and degraded, a cascade of downstream signaling disruptions occurs, leading to potent antitumor effects. One of the primary pathways affected is the AKT/PI3K/mTOR axis, which governs cell survival, growth, and metabolism. Several Hsp90–Cdc37 disruptors, including DCZ3112 and platycodin D, have been shown to suppress AKT phosphorylation, thereby dampening prosurvival signaling and sensitizing cancer cells to apoptotic cues.^44,57^ In parallel, inhibition of the ERK/MAPK pathway has been observed with compounds such as DCZ3112 and okicamelliaside, both of which markedly reduce ERK1/2 phosphorylation, impairing cell proliferation and migratory potential.^37,57^ A notable therapeutic advantage of targeting the Hsp90–Cdc37 complex is the ability to interfere with adaptive resistance mechanisms. For instance, the co-administration of platycodin D with the mTOR inhibitor everolimus effectively terminated feedback reactivation of AKT, a well-documented resistance pathway that limits the efficacy of mTOR-targeted therapies.^44^ Collectively, the coordinated suppression of these signaling cascades contributes to robust and multifaceted antitumor activity, making Hsp90–Cdc37 PPI disruption a compelling strategy for cancer treatment.

### 5.3 Induction of apoptosis and cell cycle arrest

Disruption of the Hsp90–Cdc37 interaction results in the degradation of critical kinase clients, triggering apoptotic signaling and interfering with cell cycle progression. Several inhibitors have demonstrated the ability to induce apoptosis in diverse cancer models. For example, compound 6, a celastrol-triazole derivative, significantly promoted apoptosis in MDA-MB-231 breast cancer cells, while FW-04-806 induced dose- and time-dependent apoptosis in HER2-overexpressing breast cancer lines.^48,73^ Similarly, Pep-5 and TAT-conjugated peptidomimetics were shown to elicit strong apoptotic responses in hepatocellular carcinoma and colorectal cancer models, respectively.^62,63^ In parallel, many Hsp90–Cdc37 disruptors cause arrest at specific phases of the cell cycle, thereby halting proliferation. Compound 6 was associated with G0/G1 phase arrest, reflecting disruption of early cell cycle progression.^48^ In contrast, FW-04-806 induced G2/M arrest, whereas compound 11g caused S-phase accumulation in A549 lung cancer cells, indicating inhibition of DNA synthesis or replication stress.^50,73^ Additionally, DDO-5936 induced G1-phase arrest through the downregulation of CDK4, a known client of the Hsp90–Cdc37 complex.^54^ Together, these mechanisms result in the irreversible loss of proliferative capacity, offering a dual-hit strategy of inducing cell death while halting tumor growth.

### 5.4 Overcoming drug resistance

A notable advantage of targeting the Hsp90–Cdc37 axis lies in its ability to overcome resistance mechanisms that limit the efficacy of conventional therapies. For instance, DCZ3112 was shown to overcome trastuzumab resistance in HER2-positive breast cancer, offering a promising approach in cases where HER2-targeted therapies have failed.^57^ Similarly, compound 11g reversed acquired resistance in A549 lung cancer cells, highlighting its potential to restore drug sensitivity in refractory tumors.^50^ Moreover, combination therapy using platycodin D alongside mTOR inhibitors effectively disrupted feedback survival loops—particularly the compensatory reactivation of AKT signaling—a common escape mechanism in mTOR-targeted treatments.^44^ These findings suggest that Hsp90–Cdc37 disruptors could serve as effective agents in treatment-resistant cancers, either as monotherapies or in rationally designed combination regimens.

### 5.5 Avoidance of heat shock response

A key therapeutic advantage of targeting the Hsp90–Cdc37 interface is the minimal induction of the heat shock response (HSR), which contrasts sharply with the effects of classical Hsp90 ATPase inhibitors.^1,17^ Traditional agents such as geldanamycin strongly upregulate Hsp70 and Hsp27, activating cytoprotective pathways that can promote tumor cell survival and contribute to treatment resistance.^74^ In contrast, compounds that disrupt the Hsp90–Cdc37 interaction tend to spare or only modestly activate this stress response. For example, platycodin D did not elevate Hsp70 expression, even at effective doses, while celastrol induced only a modest, 12-fold increase in Hsp70, and only at relatively high concentrations—much lower than the levels observed with classical inhibitors.^44^ Similarly, K-Ras-selective small molecules targeting the Hsp90–Cdc37 interface effectively inhibited kinase clients without triggering the HSR.^47^ Notably, compounds 8c and 13g demonstrated potent anticancer activity without upregulating heat shock proteins, further reinforcing this mechanistic distinction.^36^ By avoiding activation of the HSR, Hsp90–Cdc37 disruptors offer the potential to reduce cytoprotective feedback and delay or prevent resistance, thereby enhancing their utility as targeted cancer therapeutics.

### 5.6 *In vivo* antitumor effects

Multiple Hsp90–Cdc37 disruptors have demonstrated potent antitumor efficacy *in vivo*, further validating this therapeutic strategy beyond *in vitro* studies. DCZ3112, for instance, significantly inhibited tumor growth in BT-474 HER2-positive xenografts, including models resistant to trastuzumab, highlighting its potential in overcoming targeted therapy resistance.^57^ Similarly, FW-04-806 showed enhanced suppression of tumor progression in SKBR3 xenografts, which overexpress HER2, compared to more resistant MCF-7 models, suggesting selectivity for kinase-dependent tumor types.^73^ Celastrol, administered at 3 mg kg^−1^, achieved over 80% inhibition of tumor growth and metastasis in RIP1-Tag2 transgenic mouse models, reinforcing its broad-spectrum antitumor activity.^47^ Notably, okicamelliaside exhibited strong antitumor effects in A549 lung cancer xenografts, with minimal systemic toxicity, supporting its potential as a safe and selective Hsp90–Cdc37 PPI inhibitor.^37^ These *in vivo* findings collectively emphasize the translational promise of this class of molecules for effective cancer therapy.

## 6. Advantages of targeting the Hsp90–Cdc37 interface

Targeting the Hsp90–Cdc37 PPI offers several compelling therapeutic advantages over traditional strategies that inhibit Hsp90's ATPase activity.^5,13^ By disrupting this critical interaction selectively, these inhibitors achieve more refined modulation of chaperone activity with improved specificity, reduced systemic toxicity, and the potential to overcome resistance mechanisms often encountered in cancer therapy.^75,76^

### 6.1 Enhanced specificity for oncogenic kinases

One of the most compelling advantages of targeting the Hsp90–Cdc37 interaction is the enhanced specificity it offers for oncogenic kinases, a stark contrast to the broad inhibition profile of classical Hsp90 ATPase inhibitors.^19,77^ While Hsp90 is a ubiquitous molecular chaperone responsible for stabilizing a wide array of client proteins—including both oncogenic drivers and essential housekeeping proteins—ATP-competitive inhibitors indiscriminately block its activity.^19,77^ This lack of selectivity often leads to off-target effects in normal tissues and contributes to the toxicity observed in clinical settings. In contrast, Cdc37 functions as a kinase-specific cochaperone, recruiting a distinct subset of Hsp90 clients—primarily protein kinases, including many that drive malignant transformation and tumor progression such as Akt, Cdk4, Raf, and HER2.^13,32^ Disrupting the Hsp90–Cdc37 interface allows for preferential destabilization of these oncogenic kinase clients while largely sparing non-kinase clients.^13,18^ This targeted mechanism yields a more favorable therapeutic index and reduces collateral damage to normal cells. For instance, structure-based inhibitors such as DDO-5936 and DDO-5994 were shown to selectively degrade Cdk4 and other kinase clients in cancer cells without broadly impacting the rest of the Hsp90 proteome.^54,55^ This selective cytotoxicity toward tumor-specific signaling networks underscores the potential of Hsp90–Cdc37 disruptors to achieve cancer cell-specific inhibition with minimal systemic toxicity, representing a significant advancement in the development of safer, more precise chaperone-targeted therapies.

### 6.2 Avoidance of cytoprotective heat shock response

A well-recognized limitation of classical Hsp90 inhibitors, particularly ATPase-targeting agents like geldanamycin, is the robust induction of the heat shock response (HSR).^78,79^ This response is characterized by the upregulation of cytoprotective proteins such as Hsp70, Hsp27, and other molecular chaperones, which collectively enhance cellular stress tolerance, promote resistance to apoptosis, and ultimately narrow the therapeutic window of these agents.^78,79^ The activation of HSR acts as a compensatory mechanism, undermining the pro-apoptotic and antiproliferative effects of Hsp90 inhibition. In contrast, disruptors of the Hsp90–Cdc37 PPI offer a strategic advantage by minimizing HSR activation, thereby sustaining their antitumor efficacy without triggering protective stress responses. For instance, platycodin D has been shown to suppress oncogenic client proteins without inducing Hsp70 expression.^44^ Similarly, K-Ras-selective inhibitors and small molecules such as compounds 8c and 13g effectively reduced Hsp90-dependent kinase levels while leaving heat shock protein expression largely unaffected.^36,58^ Even celastrol, despite its broader biological activity, induced only a modest increase in Hsp70 compared to classical Hsp90 inhibitors, and only at higher concentrations.^47^ This reduced propensity to activate HSR not only enhances the cytotoxic effects of Hsp90–Cdc37 disruptors but also prevents the emergence of drug resistance associated with stress-induced survival pathways. As such, targeting the Hsp90–Cdc37 interface represents a mechanistically distinct and clinically advantageous strategy for cancer therapy.

### 6.3 Reduced systemic toxicity

Traditional Hsp90 ATPase inhibitors, while effective in degrading a broad range of client proteins, often suffer from poor therapeutic tolerability due to their pan-inhibitory action. By interfering with essential chaperone functions in normal tissues, these agents disrupt critical cellular processes and have been associated with serious off-target toxicities, including hepatotoxicity, cardiovascular side effects, and ocular damage.^80^ These adverse effects have posed significant barriers to the clinical development and dosing flexibility of ATP-competitive Hsp90 inhibitors. In contrast, disruptors of the Hsp90–Cdc37 PPI offer a more targeted approach, selectively destabilizing kinase clients—many of which are oncogenic—while largely sparing non-kinase proteins that are vital to normal cellular function.^80^ This selectivity translates into a more favorable toxicity profile. For example, DCZ3112 effectively inhibited tumor growth in HER2-positive xenograft models with minimal systemic toxicity.^57^ Similarly, FW-04-806 showed selective activity against HER2-overexpressing tumors and low toxicity in treated animals.^73^ Okicamelliaside also demonstrated high antitumor efficacy in A549 xenografts while maintaining excellent tolerability.^37^ The reduced toxicity of these agents is attributed to their focused degradation of oncogenic kinase clients, avoiding the global disturbance of protein homeostasis that characterizes pan-Hsp90 inhibition. As a result, Hsp90–Cdc37 disruptors present a promising class of safer, more selective anticancer therapeutics.

### 6.4 Overcoming resistance to conventional therapies

Resistance to targeted therapies—including kinase inhibitors and Hsp90 ATPase inhibitors—remains a significant barrier in cancer treatment. Tumor cells often adapt by activating compensatory pathways, mutating target proteins, or engaging survival mechanisms such as the heat shock response. In this context, Hsp90–Cdc37 disruptors offer a novel approach to circumvent resistance mechanisms by selectively destabilizing oncogenic kinases that are often central to resistant phenotypes. For instance, DCZ3112 demonstrated efficacy in trastuzumab-resistant HER2-positive breast cancer cells, suggesting its potential to restore sensitivity in tumors unresponsive to receptor tyrosine kinase inhibitors.^57^ Similarly, platycodin D was shown to potentiate the effect of mTOR inhibitors by blocking feedback activation of AKT, a common resistance mechanism following mTOR pathway suppression.^44^ Additionally, compound 11g successfully reversed drug resistance in A549 lung cancer cells, further emphasizing the role of Hsp90–Cdc37 disruption in overcoming acquired therapeutic resistance.^50^

### 6.5 Potential for combination therapies

Given their selective mechanism of action, Hsp90–Cdc37 disruptors are well-suited for use in combination therapy regimens. Their ability to weaken oncogenic signaling networks can enhance the efficacy of other agents while allowing for dose reduction, thereby minimizing toxicity. For example, combining platycodin D with everolimus (an mTOR inhibitor) effectively inhibited survival pathway reactivation, a frequent cause of mTOR therapy failure.^44^ Similarly, DCZ3112 has been shown to work synergistically with trastuzumab, enhancing its effect in resistant HER2-positive models.^57^ Beyond targeted agents, these disruptors may also be paired with standard chemotherapies to increase apoptosis and suppress tumor cell proliferation. Such synergistic strategies not only improve therapeutic outcomes but also expand the clinical applicability of Hsp90–Cdc37 disruptors as adjuncts in multidrug regimens.

### 6.6 Targeting dynamic hotspots in the chaperone cycle

Recent advances in computational modeling and structural biology have revealed that the Hsp90–Cdc37–kinase complex is governed by a network of dynamic and allosteric regulatory hotspots that play critical roles in coordinating chaperone activity throughout the folding cycle.^69^ These interaction nodes are not static but fluctuate in conformation, offering transient yet druggable sites for therapeutic intervention. By designing PPI disruptors that selectively target these dynamic hotspots, researchers can achieve precise interference with chaperone–client assembly at key regulatory checkpoints, without inducing global suppression of Hsp90's activity across its broad client spectrum.^81^ This approach marks a shift toward next-generation precision oncology, enabling the selective dismantling of tumor-specific signaling complexes while sparing normal cellular processes. Rather than blocking ATP hydrolysis or inhibiting all client interactions, targeting dynamic interface regions within the Hsp90–Cdc37–kinase axis allows for context-specific disruption, effectively modulating oncogenic survival pathways with greater specificity and reduced toxicity.^81^ As our understanding of these conformational landscapes deepens, it paves the way for more refined, mechanism-based cancer therapeutics.

## 7. Challenges and future directions

While targeting the Hsp90–Cdc37 PPI represents a promising therapeutic strategy, several key challenges remain that must be addressed to translate these agents from preclinical discovery into effective clinical therapies.^1,13,82^ A clear understanding of these hurdles is essential for guiding future research and development efforts.

### 7.1 Difficulties in targeting large and dynamic PPI interfaces

One of the primary challenges in developing Hsp90–Cdc37 disruptors lies in the structural nature of the PPI interface. Unlike classical enzyme active sites or receptor pockets, the Hsp90–Cdc37 binding surface is relatively large, flat, and conformationally dynamic, offering few well-defined cavities for conventional small-molecule binding.^13,23^ This makes it inherently difficult to achieve high-affinity and selective inhibition using traditional drug design approaches. Although critical contact residues such as Glu47 on Hsp90 and Arg167 on Cdc37 have been identified as functional hotspots, these sites are often transient or partially buried, complicating efforts to design compounds that can stably engage them without off-target effects.^54,55^ To address these limitations, researchers are increasingly relying on advanced computational tools, molecular dynamics simulations, and fragment-based drug discovery to map dynamic interaction regions and identify druggable sub-pockets that emerge during the chaperone cycle.^83^ Targeting these dynamic allosteric sites offers a promising strategy to achieve precise modulation of the Hsp90–Cdc37 complex while preserving overall chaperone homeostasis.^29,84^ Continued progress in these areas will be crucial to surmount the structural and kinetic complexities of this PPI interface.

### 7.2 Optimization of drug-like properties

While several Hsp90–Cdc37 PPI disruptors have demonstrated potent *in vitro* activity, many still face significant pharmacokinetic and drug development hurdles that limit their clinical potential.^40,70^ Common issues include poor aqueous solubility, limited metabolic stability, and inadequate oral bioavailability—factors that collectively hinder effective systemic delivery and therapeutic efficacy.^40,70^ For example, natural compounds like celastrol and some of its early derivatives suffer from low solubility and rapid degradation, reducing their bioavailability *in vivo*.^40,70^ To overcome these barriers, researchers have pursued various medicinal chemistry strategies. These include the development of more soluble celastrol analogs such as CEL20 and compound 41, which incorporate polar functional groups to enhance solubility and bioactivity.^40,70^ Peptide-based disruptors, like Pep-5, have been cyclized to improve proteolytic stability and membrane permeability. Similarly, hydrophobic substitutions, as seen in the development of DDO-5994, have helped optimize molecular interactions with poorly defined target pockets and improve pharmacological profiles.^62^ Despite these advances, further refinement of absorption, distribution, metabolism, and excretion (ADME) properties remains essential for successful clinical translation of these promising agents.

### 7.3 *In vivo* efficacy and safety validation

Several Hsp90–Cdc37 disruptors, including DCZ3112, celastrol, and FW-04-806, have demonstrated promising *in vivo* antitumor efficacy in xenograft models, underscoring their potential as next-generation cancer therapeutics.^47,57,73^ However, despite these encouraging preclinical results, comprehensive pharmacokinetic (PK) profiling for many of these compounds remains limited. Critical data on bioavailability, tissue distribution, clearance, and metabolic stability are often incomplete, making it difficult to fully assess their translational potential. Moreover, long-term safety studies are needed to evaluate possible off-target toxicities, especially with chronic dosing regimens. Another emerging area of interest is the immune-modulatory effect of these agents, including their potential impact on the tumor microenvironment. Given the growing relevance of immuno-oncology, understanding whether Hsp90–Cdc37 disruptors modulate immune infiltration, checkpoint expression, or cytokine signaling will be important for designing combination regimens and maximizing therapeutic benefit. Future studies should focus on expanded animal model validation, chronic toxicity assessments, and immune profiling, particularly in the context of combination therapies.^47,57,73^

### 7.4 Resistance mechanisms

While Hsp90–Cdc37 disruptors have shown the ability to overcome resistance to ATPase inhibitors and kinase-targeted therapies, the potential for resistance to these disruptors themselves is an important and largely uncharacterized challenge.^85^ Tumor cells may adapt by upregulating alternative cochaperones (*e.g.*, Hsp70, Hsp27), activating parallel survival pathways such as PI3K/AKT, ERK/MAPK, or mTOR, or acquiring mutations at the Hsp90–Cdc37 interface that reduce drug binding without impairing complex function.^86,87^ These adaptive responses could diminish the efficacy of PPI-targeted strategies over time. Therefore, longitudinal studies that monitor resistance evolution during prolonged treatment are crucial. Additionally, rational combination strategies—for example, pairing Hsp90–Cdc37 disruptors with AKT, ERK, or mTOR inhibitors—may preempt or overcome resistance by simultaneously blocking compensatory escape routes.^44,57,58^ Such integrated approaches will be key to sustaining durable responses and advancing these agents toward clinical application.

### 7.5 Peptide and peptidomimetic challenges

Peptide-based inhibitors targeting the Hsp90–Cdc37 interface offer the advantage of high binding specificity due to their ability to mimic native PPIs.^88^ However, they face several well-documented limitations that hinder their clinical translation. These include poor stability in plasma, rapid enzymatic degradation by proteases, and limited cell membrane permeability, which collectively compromise their systemic bioavailability and therapeutic efficacy.^88^ Recent innovations—such as TAT-conjugated peptides like TAT-DDO-59120 and cyclic peptidomimetics—have demonstrated improved cellular uptake and resistance to degradation.^62^ Nonetheless, further optimization is needed. Strategies such as peptide stapling, PEGylation, or encapsulation using nanoparticle-based delivery systems may enhance *in vivo* stability and target specificity, paving the way for the broader application of peptide-based disruptors in oncology.

### 7.6 Need for biomarker development

A critical barrier to the clinical advancement of Hsp90–Cdc37 disruptors is the lack of validated biomarkers to guide patient selection and monitor therapeutic responses.^89^ Predictive biomarkers are essential for identifying patients most likely to benefit from these agents, while pharmacodynamic markers are needed to evaluate target engagement and efficacy during treatment. Potential candidates include the levels of degraded kinase clients such as Akt, HER2, and Cdk4, as well as the activation status of downstream signaling pathways, including p-AKT and p-ERK.^1^ Additionally, quantifying Hsp90–Cdc37 complex abundance through immunoprecipitation or proximity ligation assays may serve as a direct measure of drug action. The development of non-invasive imaging modalities or blood-based assays to track these biomarkers would greatly facilitate early-phase clinical trials and support the implementation of personalized therapy strategies in precision oncology.

### 7.7 Future perspectives

The future of Hsp90–Cdc37 disruptor development lies in a multidisciplinary, integrated approach that leverages insights from structural biology, computational modeling, and systems pharmacology.^13,90^ One promising avenue is the design of dual-function molecules that combine PPI disruption with mild ATPase modulation, potentially achieving synergistic inhibition while minimizing toxicity.^5^ Efforts to design allosteric modulators that bind to distant regulatory sites and destabilize the Hsp90–Cdc37 complex indirectly are also gaining traction.^13,18^ Furthermore, combining Hsp90–Cdc37 disruptors with immune checkpoint inhibitors may enhance tumor immunogenicity, offering novel immuno-oncological applications.^91,92^ Advances in artificial intelligence (AI) and machine learning (ML) are expected to accelerate the discovery and optimization of next-generation disruptors by enabling predictive modeling of dynamic interaction hotspots.^93,94^ In parallel, nanoformulation strategies hold promise for improving the delivery and bioavailability of hydrophobic natural products like celastrol and withaferin A, which have shown efficacy but suffer from poor solubility.^95^ Ultimately, the integration of medicinal chemistry, computational drug design, biomarker development, and preclinical validation will be critical to overcoming current limitations and translating Hsp90–Cdc37 PPI inhibitors into effective cancer therapeutics.

## 8. Conclusion

The Hsp90–Cdc37 chaperone complex plays a pivotal role in the stabilization and maturation of a large subset of oncogenic kinases, supporting tumor growth, survival, and resistance to therapy. Traditional strategies to inhibit Hsp90 by targeting its ATPase activity have been hampered by limited specificity, induction of cytoprotective heat shock responses, and systemic toxicity, ultimately restricting their clinical success. Disrupting the specific PPI between Hsp90 and Cdc37 has emerged as a highly promising alternative approach. By selectively destabilizing oncogenic kinase clients without broadly impairing the entire Hsp90 chaperone system, Hsp90–Cdc37 disruptors offer distinct advantages: enhanced specificity for malignant cells, reduced activation of heat shock responses, improved safety profiles, and the ability to overcome resistance mechanisms common in kinase-driven cancers.

A wide range of small molecules, natural products, and rationally designed peptides and peptidomimetics have demonstrated the ability to disrupt the Hsp90–Cdc37 interaction. Compounds such as DCZ3112, DDO-5994, celastrol derivatives, platycodin D, okicamelliaside, and engineered peptides like Pep-5 and TAT-DDO-59120 highlight the diverse chemical and biological strategies employed to achieve effective disruption. These agents have shown potent *in vitro* and *in vivo* antitumor activities, suppression of key signaling pathways (AKT, ERK, HER2), induction of apoptosis, and the ability to enhance or restore sensitivity to other anticancer therapies. Nonetheless, challenges remain. The dynamic and shallow nature of the Hsp90–Cdc37 interface, optimization of drug-like properties, *in vivo* stability, and the development of reliable predictive biomarkers are critical areas that require further research. Additionally, understanding the potential for acquired resistance to Hsp90–Cdc37 disruptors and refining delivery methods for peptide-based agents are essential for clinical translation.

Future directions will likely involve the integration of computational modeling, artificial intelligence-driven drug design, advanced nanodelivery systems, and combination therapeutic approaches to fully exploit the therapeutic window offered by Hsp90–Cdc37 disruption. The rational design of dual-function inhibitors, capable of simultaneously disrupting multiple oncogenic survival mechanisms, also holds great promise. In conclusion, targeting the Hsp90–Cdc37 interface represents a paradigm shift in the field of molecular chaperone-based therapies. By combining precision, specificity, and reduced toxicity, this emerging class of agents offers a powerful and versatile strategy for the treatment of a wide range of human malignancies. Continued research and multidisciplinary collaboration will be crucial to translating these advances from bench to bedside, paving the way for next-generation precision oncology therapeutics.

## Data availability

No primary research results, software or code have been included and no new data were generated or analyzed as part of this review.

## Conflicts of interest

Authors declare no conflict of interest
